# Human Serum Albumin Conjugates of 7-Ethyl-10-hydroxycamptothecin (SN38) for Cancer Treatment

**DOI:** 10.1155/2014/963507

**Published:** 2014-05-07

**Authors:** Nima Sepehri, Hasti Rouhani, Ahmad Reza Ghanbarpour, Mehdi Gharghabi, Faranak Tavassolian, Mohsen Amini, Seyed Nasser Ostad, Mohammad Hossein Ghahremani, Rassoul Dinarvand

**Affiliations:** ^1^Department of Pharmaceutics, Faculty of Pharmacy, Tehran University of Medical Sciences, P.O. Box 14155-6451, Tehran, Iran; ^2^Nanotechnology Research Center, Faculty of Pharmacy, Tehran University of Medical Sciences, P.O. Box 14155-6451, Tehran, Iran; ^3^Nano Alvand Co., Avicenna Tech. Park, Tehran University of Medical Sciences, Tehran, Iran; ^4^Department of Toxicology-Pharmacology, Faculty of Pharmacy, Tehran University of Medical Sciences, Tehran, Iran; ^5^Department of Medicinal Chemistry, Faculty of Pharmacy, Tehran University of Medical Sciences, Tehran, Iran

## Abstract

SN38 (7-ethyl-10-hydroxy-comptothecin) is a potent metabolite of irinotecan, which has been approved for treatment of metastatic colorectal cancer. Considering the notable potency of SN38, it has been introduced as an anticancer candidate. In this study, human serum albumin (HSA) conjugates of SN38 were formulated to overcome the solubility problem beside improving the active form stability and tumor tissue targeting. In this target, two different molar ratios of conjugates (SN38 : HSA 15 : 1 and 60 : 1) were prepared by derivatization of 20-hydroxyl group of SN38 with glycine, followed by addition of succinyl group to glycine through which HSA was covalently attached. The conjugates with particle size of about 100 nm revealed enhanced water solubility and were relatively stable in neutral and acidic solutions. For SN38-HSA-15 and SN38-HSA-60 IC_50_ values were compared with irinotecan in HT-29 human colon cancer cells. Furthermore, biodistribution studies of SN38-HSA conjugate resulted in proper blood concentration level within 4 h. Moreover, blood cytotoxicity assay revealed no toxicity effect on liver and spleen. Collectively, our present investigation offers a water-soluble form of SN38 attached to HSA and suggests using favorable properties as a promising anticancer agent for further preclinical and clinical investigations.

## 1. Introduction

SN38 (7-ethyl-10-hydroxy-comptothecin) belongs to the 20 (s)-camptothecin groups which are known as potent topoisomerase I inhibitors, a key enzyme being involved in DNA replication and transcription processes in certain steps of the cell cycle. The effectiveness of SN38 as a possible treatment approach has been explored in a variety of human cancers, including colorectal, lung, and ovarian as well [1–3]. Previous cytotoxic studies have corroborated that SN38 is 100-fold more potent than irinotecan (camptothecin-11). Irinotecan has FDA approval for the treatment of metastatic colorectal cancer [4, 5]. Following irinotecan administration, a few percentage of irinotecan converted to the active SN38 via carboxylesterase mediated cleavage in the liver. This metabolic conversion is relatively depending upon genetic variability. Thus, SN38 itself can be introduced as a promising anticancer candidate. Despite the fact that SN38 has efficacious activity toward tumor cells, drawbacks remain with its clinical application due to its extremely poor solubility in aqueous solution and other pharmaceutically acceptable solvents [6, 7]. In this regard, Zhao et al. in 2008 introduced poly (ethylene glycol)-SN38 as a water-soluble conjugate [8]. Moreover, multiple solid tumors were employed as a preclinical model in Sapra et al. in 2008 [7] and 2009 [9] studies. The results showed that PEG-SN38 conjugates demonstrated a significantly enhanced therapeutic index compared to that of irinotecan. These studies were carried out in completion of the previous studies by Conover et al. in 1997 [10] and 1998 [11] in which, PEG conjugated camptothecin was designed and its antitumor efficacy was assessed.

Active form of SN38 has a closed lactone ring which can be converted to an inactive carboxylate form at physiological pH [12]. Hence, developing a certain formulation in which the active lactone form of SN38 is maintained besides promoting the solubility is crucial for the achievement of clinical efficacy which has been considered as the main purpose of various investigations [8, 13–17]. Irrespective of the solubility issue and stability of active form, increasing tumor targeting ability and development of control drug delivery of SN-38 still remain as an ongoing debate thus far [18, 19].

Human serum albumin (HSA) is a nontoxic, biocompatible, and biodegradable macromolecule, being regarded as the most abundant protein in blood serum. Additionally, the physical robust properties of HSA coupled with its preferential uptake in tumor tissue make it an ideal carrier for drug delivery. It has been shown that albumin markedly accumulates in tumor tissues due to leaky capillary combined with a defective lymphatic drainage system in tumor interstitium which is known as enhanced permeation and retention (EPR) effect [20–22]. In addition, conjugation with macromolecules such as albumin can improve drug pharmacokinetic profile due to the long half-life of albumin in the body [23]. Another advantage of albumin conjugation is overcoming multidrug resistance against anticancer drugs [24]. Based on these findings, development of drug conjugates using albumin has been considered as a possible approach to enhance drug targeting.

Yao et al. in 2013 synthesized SN38 conjugate with bovine serum albumin (BSA) in which SN38 was covalently attached to the only free sulfhydryl group at cysteine on BSA. In their method, BSA and SN38 were conjugated in molar ratio value of 1 : 1 [25].

Since drug loading is highly important concern for clinical application, in this study our main goal is HSA conjugation of SN38 with higher molar ratio. Water-soluble conjugations of SN38 with HSA have been prepared by derivatization of 20-hydroxyl group of SN38 with glycine, followed by addition of succinyl group to glycine through which HSA was covalently attached. The characteristics of conjugations were evaluated for their size, zeta potential, drug content, morphology, and* in vitro* stability. The* in vitro* cytotoxicity of conjugates against HT-29 human colon cancer cell line was reported in comparison with free SN38 and irinotecan. Additionally, the* in vivo* biodistribution and blood cytotoxicity were further reported.

## 2. Methods and Materials

### 2.1. Materials

SN38 was purchased from Knowshine Co. (China). Anhydrous pyridine, di-tert-butyl dicarbonate, 4-dimethylaminopyridine (DMAP), trifluoroacetic acid (TFA), succinic anhydride, N,N′-dicyclohexylcarbodiimide (DCC), 1-ethyl-3-(3-dimethylaminopropyl) carbodiimide (EDC), triethyl amine (TEA), anisole, and used solvents were purchased from Merck (Darmstadt, Germany). BOC-glycine, N-hydroxy-sulfo-succinimide (sulfo-NHS), human serum albumin (HSA), and 3-(4,5-dimethylthiazol-2-yl)-2,5-diphenyl tetrazolium bromide (MTT) were obtained from Sigma-Aldrich (St. Louis, MO, USA). Roswell Park Memorial Institute medium (RPMI 1640), Fetal Bovine Serum (FBS), penicillin, and streptomycin were provided from Life technologies (Grand Island, NY, USA). HT-29 human colon cancer cell line was obtained from American Type Culture Collection (ATCC).

### 2.2. Conjugates Preparation

**Figure 1:**
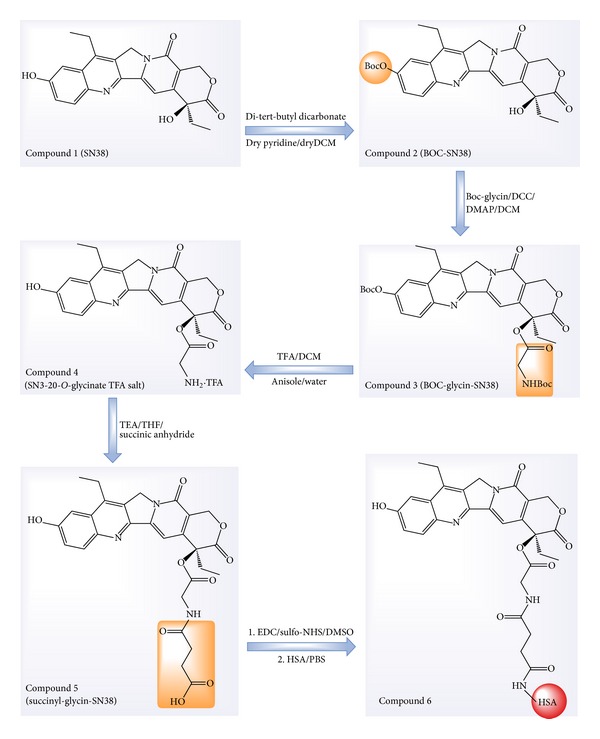
SN38-HSA synthesis scheme. The process consists of phenolic OH protection of SN38 (Compound 2), addition of glycine molecule (Compound 3), OH deprotection (Compound 4), succinyl addition (Compound 5), and HSA conjugation (Compound 6).

The overall scheme of SN38 conjugation to HSA was represented in Figure 1. This process was carried out in four major steps: (1) protection of phenolic OH group of SN38 by di-tert-butyl dicarbonate, (2) derivatization of aliphatic OH group of SN38, (3) preparation of succinyl-glycine derivative, and (4) conjugation with HSA. Detailed procedures for each step are described as follows.

#### 2.2.1. Protection of the Phenolic OH Group (Compound 2)

As shown in Figure 1, Compound 2 was synthesized according to the previously reported literature method [8]. SN38 (300 mg) was added to di-tert-butyl dicarbonate (0.5 mL) in the presence of anhydrous dichloromethane (DCM, 150 mL) and anhydrous pyridine (1.5 mL). The mixture was stirred afterwards at room temperature. After 24 h, the solution was filtered and was washed thereafter three times with HCl (0.01 N). The organic phase was separated by addition of sodium sulfate and was evaporated under reduced pressure to obtain di-tert-butyl dicarbonate-SN38 (BOC-SN38) (93% yield).

#### 2.2.2. Derivatization of Aliphatic OH Group of SN38 (Compounds 3 and 4)

Compound 3 was prepared based upon the previous reported procedure [7, 16] with some modifications. Briefly, BOC-SN38 from previous step (322 mg) was dissolved in DCM (10 mL) and BOC-glycine (230 mg), N,N′-dicyclohexylcarbodiimide (DCC) (270 mg), and 4-dimethylaminopyridine (DMAP) (96 mg) were added under stirring. The reaction was accomplished for 12 h at room temperature. The mixture was then filtered and Compound 3 was isolated using flash chromatography (yellow powder, %92 yield). Deprotection of Compound 3 was carried out by adding it to a mixture consisting of 2 mL of trifluoroacetic acid (TFA), 0.5 mL of DCM, 0.12 mL of anisole, and 0.06 mL of water. The mixture was stirred for 30 minutes at ambient temperature. SN38-20-O-glycinate TFA salt (Compound 4) was yielded by addition of diethyl ether.

#### 2.2.3. Preparation of Succinyl-Glycine Derivative (Compound 5)

Triethyl amine (TEA) (0.16 mL) and succinic anhydride (117 mg) were added to a solution of SN38-20-O-glycineate TFA salt (330 mg) in 10 mL of anhydrous tetrahydrofuran (THF). The solution was stirred at room temperature overnight and filtered afterwards. The solvent was evaporated under reduced pressure. The product was then washed with HCl (0.001 N) and the precipitate was further purified by flash chromatography to obtain compound 5.

#### 2.2.4. Conjugation with HAS (Compound 6)

The conjugation with HSA was achieved according to a reported literature method [26, 27]. 1-Ethyl-3-(3-dimethylaminopropyl) carbodiimide (EDC) (34 mg) and N-hydroxy-sulfo-succinimide (sulfo-NHS) (39 mg) were added to a mixture of compound 5 (50 mg) in anhydrous dimethyl sulfoxide (DMSO) (5 mL) for 5 h under stirring at ambient temperature. The HSA solution was prepared by addition of HSA in a suitable amount of phosphate buffered solution (PBS 0.1 M, pH 7.3 plus 50 mM NaCl). HSA solution was added to the solution from the previous step containing 15 and 60 times molar excess of SN38 attached to linker. The reaction was maintained under stirring overnight in ambient temperature. After centrifugation the mixture was dialyzed against deionized water for 24 h. The conjugates were further lyophilized for storage.

### 2.3. Characterization of the Conjugates

#### 2.3.1. Size, Size Distribution, and Zeta Potential

Particle size and size distribution were indicated by polydispersity index (PDI) and zeta potential of the conjugates was determined by laser light scattering (Malvern Zetasizer ZS, Worcestershire, UK). The samples were prepared by suspending the lyophilized conjugates in 10 mL of deionized water (10 *μ*g/mL). The mean value of three samples was finally reported.

#### 2.3.2. Drug Content

The drug loading of the conjugates was determined by UV-spectrophotometery method (Cecil 9000, Cecil Instruments Ltd, Cambridge, UK) via measurement of absorbance at 383 nm [28–30]. Calibration curve was obtained from six different concentrations of free SN38 ranging from 5 to 20 *μ*g/mL. Sample solution was prepared by dissolving the conjugates in methanol containing %1 trichloroacetic acid (TCA) (0.5 mg/mL). SN38 quantification in sample solution was measured using a calibration curve and the percentage of SN38 amount (mg) in conjugate was calculated as drug content.

#### 2.3.3. Scanning Electron Microscopy (SEM)

Solution of conjugate in water was spread on aluminum stub and was allowed to be dried afterwards. The samples were then coated with a thin layer of gold using the sputter coater and examined by scanning electron microscopy (SEM) (S4160, Hitachi, Japan).

### 2.4. *In Vitro* Drug Stability

3 mg of the conjugates was transferred to vessels of dissolution apparatus II-Paddle (G. B. Caleva, UK). The vessels contained 250 mL of PBS with two different pH values of 7.4 and 5.2 to simulate physiologic media and the lower pH condition of cancer cells, respectively. The medium was maintained at 37°C while being stirred by paddles at 100 rpm. At designated times, 5 mL of the medium was sampled from the vessels and the equal volume of withdrawn solution was replaced with fresh PBS. The samples were treated with 1% zinc sulfate in methanol solution to precipitate the albumin. After centrifugation (5000 g  × 10 minutes), the remaining solutions were used for further analyses. The amount of released drug was calculated by measuring fluorescent light emitted from SN38 in the solution via spectrofluorometer [31] (Shimadzu RF-5000, Japan) at *λ*_Em_ = 530 nm and *λ*_Ex_ = 375 nm and interpolating the response in the calibration curve plotted by 6 different concentrations range of 0.012 to 0.1 *μ*M. The study was performed at least in three separate experiments and the mean value was reported.

In order to investigate the stability of the conjugate in plasma, lyophilized conjugates in 0.12 mg/mL concentration were prepared in 3 mL of rat plasma. The samples were kept at 37°C, under mild stirring. At scheduled times, the whole volume of sample was taken and SN38 was extracted from plasma by adding ZnSO_4_ (2%, w/v, in mixture of 50 : 50 methanol/water) at a ratio of 1 : 2 and analyzed by above spectrofluorometer method. Standard curves were prepared by the addition of SN38 in plasma following the same process. Each experiment was repeated in triplicate [26].

### 2.5. SDS-PAGE Analysis

In order to evaluate the molecular weight (MW) of conjugates, sodium dodecyl sulfate polyacrylamide gel electrophoresis (SDS-PAGE) was performed for both conjugate forms and HSA. The gel was run under nonreducing conditions. 30 *μ*L of protein samples (HAS and SN38-HSA with two different ratios) containing about 20 *μ*g protein as well as standard ladder were loaded on 10% polyacrylamide gel. Subsequently, the gel was subjected to electrophoresis (Bio-Rad, USA) at a constant voltage of 120 V for 3 h followed by Coomassie blue staining.

### 2.6. *In Vitro* Cell Viability

HT-29 human colon cancer cell line was seeded in RPMI 1640 medium supplemented with 10% fetal bovine saline (FBS) and 1% penicillin-streptomycin at 37°C in a humidified atmosphere of 5% CO_2_. Concisely, cells were seeded in 96-well plates and allowed to reach 70% of population confluence prior to treatment procedure. Afterwards cells were incubated with either SN38 (stock solution in DMSO diluted with culture media) or SN38-HSA conjugates solution at a designated equivalent SN38 concentration ranging from 0.01 to 12.8 *μ*M or irinotecan with concentration ranging from 1 to 50 *μ*M. After 48 h, 20 *μ*L MTT solution (5 mg per mL in PBS) was added to each well, and the culture medium containing MTT solution was removed after 3 to 4 h. The formazan crystals were dissolved in 60 *μ*L DMSO and the relative absorbance was measured by a plate reader (BioTek, ELx800, VT, USA) at 590 nm followed by background correction at 690 nm. Cell viability was calculated using the following equation:

(1) $$\begin{array}{c} \text{Cell}\;\;\text{viability}\;\left(\%\right)=\left(\frac{{\text{Int}}_{\text{sample}}}{{\text{Int}}_{\text{control}}}\right)\times 100, \end{array}$$

where the Int_sample_ and Int_control_ are the colorimetric intensity of the treated cells and the control group, respectively. DMSO was utilized as vehicle agent just as the same concentration of SN38 so that the final concentration did not exceed 0.5% of the total volume. The untreated cells were regarded as control and vehicle group. Calculating IC_50_, the concentration of the drug at which 50% of cell growth is inhibited was performed by the curve fitting of the cell viability data.

### 2.7. *In Vivo* Studies

#### 2.7.1. Animal

*In vivo* studies were performed on balb/c mice with a body weight between 25 and 30 g (provided by animal care center, Faculty of Pharmacy, Tehran University of Medical Sciences). Animal experiments were approved by the ethical committee of Pharmaceutical Research Centre, Tehran University of Medical Sciences.

#### 2.7.2. Biodistribution Assay

The investigated mice were divided into three discrete groups including 5 mice in each one. The first group was treated with 150 *μ*L of free SN38 (in NaOH 0.2 N diluted with NaCl 0.9%) and the second group received SN38-HSA in NaCl 0.9% (2.5 *μ*g/kg, 150 *μ*L/injection). The third group just received NaCl 0.9% as control group. The mice were kept fasted but they had free access to water. After 4 h, mice were sacrificed and their organs including liver, spleen, heart, kidney, intestine, and lung were collected. Concomitantly, blood samples were drawn from the inferior vena cava. Subsequent to washing the tissues with PBS, they were weighted accurately and were homogenized in 3 mL PBS. The resulting homogenates as well as blood samples were centrifuged (5000 ×g, 10 min) to remove debris. Methanol was added in equal volume as that of the supernatant and was centrifuged thereafter at 5000 ×g for 10 min. The resulting samples were analyzed for fluorescent intensity by spectrofluorophotometry (Shimadzu, Japan) at *λ*_Ex_ = 379 nm and *λ*_Em_ = 531 nm. Calibration curve of SN38 from six different concentrations was obtained in a range of 0.012 to 0.1 *μ*g/mL for calculations process. The resultant data were normalized with the control group [32–35].

#### 2.7.3. *In Vivo* Blood Cytotoxicity Tests

The hematotoxicity was studied on two groups having five mice per group. SN38-HSA was dissolved in normal saline 0.9% and administered intravenously into the tail vein (150 *μ*L of solution containing 2.5 mg of SN38 per Kg body weight). The control group only received normal saline 0.9%. The mice were kept fasted while they had free access to water. 24 and 48 h later, the mice were sacrificed and blood samples were provided from the inferior vena cava. Blood cells counts by means of red blood cell (RBC), white blood cell (WBC), and hemoglobin (Hb) were carried out on a part of blood samples collected in EDTA coated tubes. The other part of blood samples was centrifuged and the plasma was used for biochemistry assays (aspartate aminotransferase (AST), alanine aminotransferase (ALT), lactate dehydrogenase (LDH), blood urea nitrogen (BUN), and creatinine (Cr)). These tests were executed by routine clinical laboratory tests.

## 3. Results

### 3.1. Preparation of HSA-SN38 Conjugates

In order to conjugate HSA with SN38, the aliphatic hydroxyl group of SN38 was derivatized. Products of each synthesis step were confirmed by ^1^H-NMR spectroscopy. In the first step, the highly reactive hydroxyl group of phenolic ring was protected by a BOC group.

^1^H-NMR (500 MHz, CDCl_3_): *δ* 8.24 (1H, d, *J* = 9.1 Hz, H7), 7.88 (1H, s, H10), 7.66 (1H, d, *J* = 9.1 Hz, H8), 7.65 (1H, s, H5), 5.73 (1H, d, *J* = 16.5 Hz, H12), 5.30 (1H, d, *J* = 16.5 Hz, H12), 5.25 (2H, s, OCH_2_-C1), 3.15 (2H, q, *J* = 7.7 Hz, CH_3_-CH_2_-C11), 1.80–1.97 (2H, m, CH_3_-CH_2_-C4), 1.61 (9H, s, t-butyl), 1.40 (3H, t, *J* = 7.7 Hz, CH_3_-CH_2_-C11), 1.02 (3H, t, *J* = 7.3 Hz, CH_3_-CH_2_-C4).

The attachment of BOC group was confirmed by an additional singlet peak at 1.61 ppm (9 H) corresponded to the three methyl groups of the BOC group.

In the second step, BOC-glycine was linked with aliphatic OH terminal of SN38 by an esteric bond. Subsequent to purification process, SN38-glycineate compound was treated with TFA to remove BOC groups, resulting in the TFA salt of the yielded compound.

^1^H-NMR (500 MHz, DMSO-d6): *δ* 8.34 (1H, s, H10), 8.01 (1H, d, *J* = 9.5 Hz, H7), 7.42 (1H, d, *J* = 8.0 Hz, H8), 7.17 (1H, s, H5), 5.73 (1H, d, *J* = 16.5 Hz, H12), 5.54 (2H, s, OCH_2_-C1), 5.32 (1H, d, *J* = 16.5 Hz, H12), 4.34 (1H, d, *J* = 17.75 Hz, CH_2_-NH_2_), 4.11 (1H, d, *J* = 17.75 Hz, CH_2_-NH_2_), 3.10 (2H, q, *J* = 7.5 Hz, CH_3_-CH_2_-C11), 2.14–2.22 (2H, m, CH_3_-CH_2_-C4), 1.30 (3H, t, *J* = 7.5 Hz, CH_3_-CH_2_-C11), 0.95 (3H, t, *J* = 7.2 Hz, CH_3_-CH_2_-C4).

The signals at 4.11 and 4.34 (as separate doublets, due to the chiral nature of the compound) indicated the two hydrogens attached to the carbon atom of the glycine linked with SN38.

The next step was formation of ester group on the linker, which was highly reactive to attack HSA nucleophilic groups (i.e., the lysyl amino terminus). Along this, succinyl group was added to the amine terminus of glycine.

^1^H-NMR (500 MHz, DMSO-d6): *δ* 8.34 (1H, s, H10), 8.01 (1H, d, *J* = 9.5 Hz, H7), 7.42 (1H, d, *J* = 8.0 Hz, H8), 7.17 (1H, s, H5), 5.73 (1H, d, *J* = 16.5 Hz, H12), 5.54 (2H, s, OCH_2_-C1), 5.32 (1H, d, *J* = 16.5 Hz, H12), 4.34 (1H, d, *J* = 17.75 Hz, CH_2_-NH_2_), 4.11 (1H, d, *J* = 17.75 Hz, CH_2_-NH_2_), 3.10 (2H, q, *J* = 7.5 Hz, CH_3_-CH_2_-C11), 2.8 (4H, m, CH_2_-SUC), 2.14–2.22 (2H, m, CH_3_-CH_2_-C4), 1.30 (3H, t, *J* = 7.5 Hz, CH_3_-CH_2_-C11), 0.95 (3H, t, *J* = 7.2 Hz, CH_3_-CH_2_-C4).

The attachment of the succinyl group was confirmed by the signals at 2.14–2.22. Such signals can be related to methyl groups of the succinyl group which have appeared as multiplet peaks. Consequently, a highly active sulfo-NHS-succinyl-SN38 was formed. Finally, conjugation between HSA and SN38 was achieved via the succinyl spacer group (Figure 1). Two different molar proportions of SN38-HSA conjugates were prepared: SN38-HSA-15 and SN38-HSA-60 in which the molar ratio of SN38 added to HSA was 15 : 1 and 60 : 1, respectively.

### 3.2. Conjugates Characterization

As depicted in Table 1, both formulations revealed the particle size of approximately 100 nm with acceptable size distribution. The average particle size of the conjugates with different molar ratios was not varied significantly, indicating that raising the molar ratio up to 60-fold did not alter the size of the conjugates. The zeta potential measurement demonstrated higher negative charge in a conjugate with higher synthesis molar ratio of SN38.

The percentage of drug loading (%w/w) on HSA for two different synthesis molar ratios was determined via UV spectrophotometry method. Owing to different solubility characteristics of SN38 and HSA in one hand and in order to remove possible shifting of absorbance wavelengths on the other hand, nonaqueous solvent medium such as methanol plus %1 TCA was used. TCA inhibits albumin precipitation in methanol while allowing valid measurement via the UV spectrophotometery [36].

The concentration of SN38 in each sample was calculated by calibration curve. Subsequently, the percentage of SN38 weight in conjugates was determined. Drug loading was 1.8 ± 0.09 and 4.4 ± 0.14%w/w for SN38-HSA-15 and SN38-HSA-60, respectively. Increase in molar ratio of SN38 augmented drug loading, which indicated that more molecules of SN38 were attached to a single molecule of HSA. However, the elevation of lipophilic drug attachment to HSA reduced the water solubility of conjugate.

**Figure 2:**
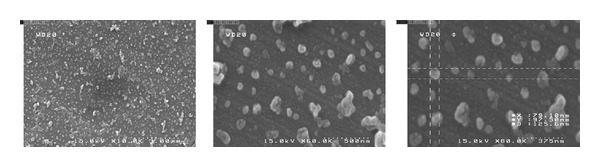
The SEM micrographs of the conjugate (SN38-HSA) in different magnification levels.

Furthermore, morphological observation of nanostructures was performed using SEM. The images represented spherical nanoparticles (NPs) having a rough surface and particle size of about 100 nm with a relatively uniform particle size distribution.  Figure 2 illustrated the appearance of the NPs in three levels of magnification.

### 3.3. *In Vitro* Drug Stability

**Figure 3:**
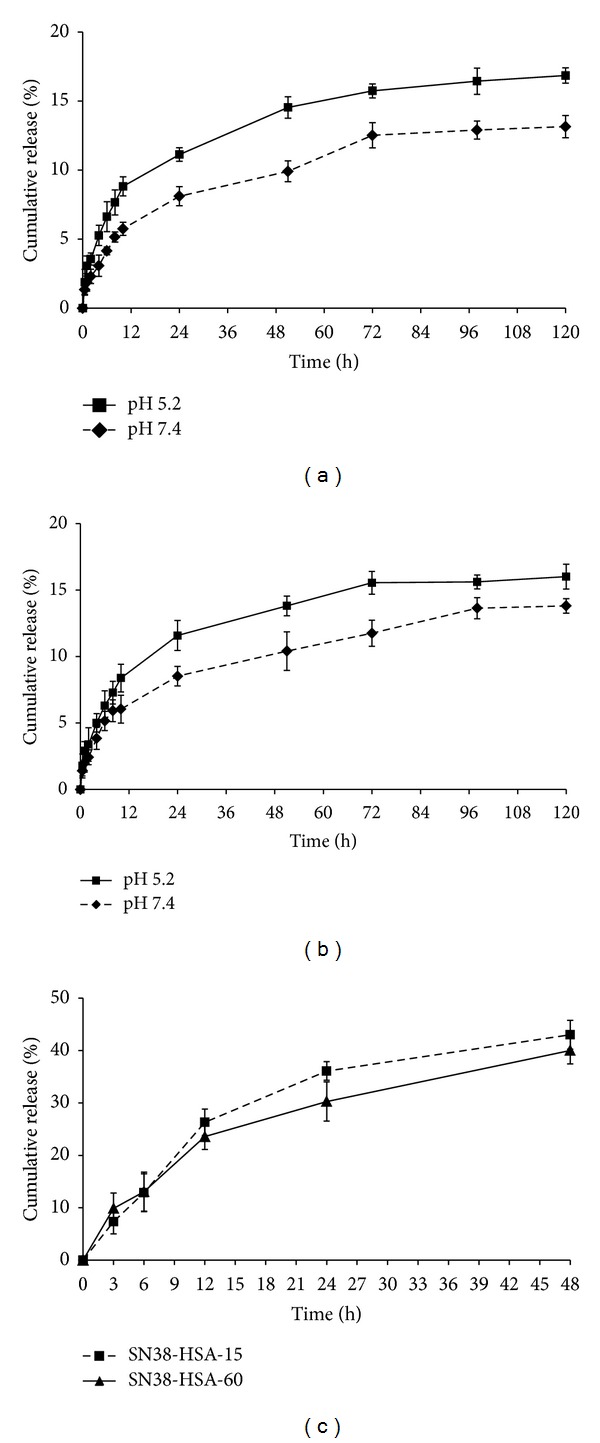
The release behavior of SN38 from (a) SN38-HSA-15 and (b) SN38-HSA-60 conjugates in pH 7.4 (◆) and 5.2 (■) and (c) SN38-HSA-15 (■) and SN38-HSA-60 (▲) conjugates in rat plasma (*n* = 3).

The drug release process was tracked up to 120 h. Figures 3(a) and 3(b) show the release profile of the two conjugate forms (SN38-HSA-15 and SN38-HSA-60) in acidic (pH 5.2) and physiologic (pH 7.4) media. For first 12 h in physiological status, SN38 release was 5.75% ± 0.480 and 6.04% ± 1.049 for SN38-HSA-15 and SN38-HSA-60, respectively. Rapid release phase is followed by a slow release rate even after 120 h that reached up to 13.15% ± 0.805 and 13.81% ± 0.540 for SN38-HSA-15 and SN38-HSA-60, respectively. Despite the higher amount of released drug in the acidic condition, the release profiles of the conjugates were similar to the physiological circumstance. Moreover, almost 8% of SN38 was released after 10 h and reached up to around 16% after 120 h for both conjugates. Thus it appears that the conjugates are reasonably stable. Overall, the results signify that SN38 can be released from the conjugates under neutral conditions even without the need for presence of a hydrolytic enzyme.

Figure 3(c) also shows that in plasma the SN38-HSA conjugates undergo rapid ester bond hydrolysis and degradation, which are reflected in the release of about 40% of SN38 from the conjugates in 48 h.

### 3.4. SDS-PAGE Analysis

**Figure 4:**
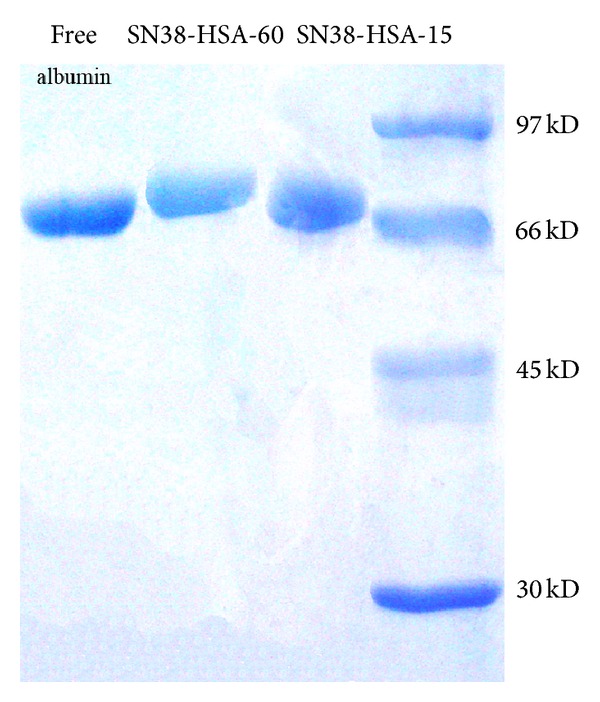
SDS-PAGE analysis of free HAS and SN38 conjugates (SN38-HSA-60 and SN38-HSA-15).

The molecular weight of conjugates was compared by using SDS-PAGE under nonreducing conditions. As shown in Figure 4, the MW of conjugates (SN38-HSA-15 and SN38-HSA-60) was approximately the same as HSA revealing that SN38-HAS has not aggregated during conjugation process. Furthermore, maintaining almost the same structure as HSA suggesting that the conjugation affected neither the molecular weight nor the global charge of HSA as a protein carrier.

### 3.5. *In Vitro* Cell Viability

**Figure 5:**
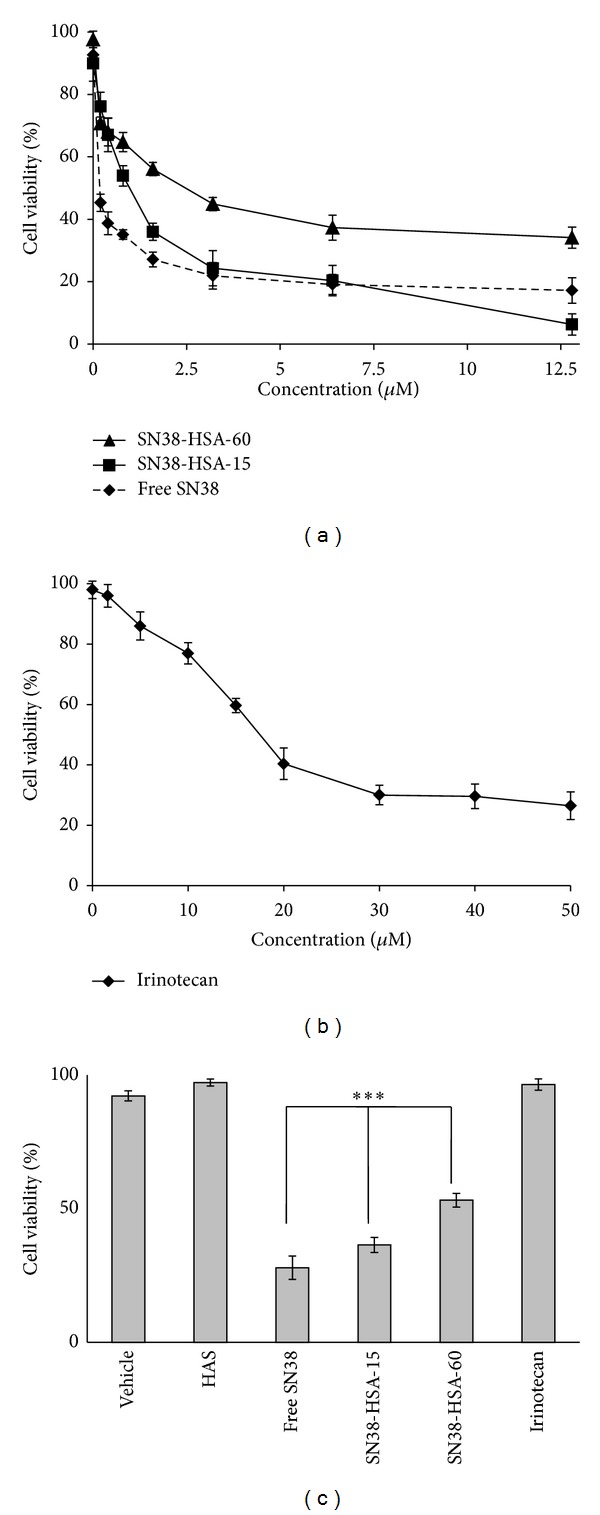
*In vitro* cytotoxicity effect on HT-29 cell line after 48 h incubation time of (a) conjugates and free SN38 with different concentrations. Symbols: ■ as SN38-HSA-15, ▲ as SN38-HSA-60, and ◆ as free SN38. (b) Irinotecan with different concentrations. (c)* In vitro* cytotoxicity of free SN38, SN38-HSA conjugates at 1.6 SN38 equivalent concentration and irinotecan (1.6 *μ*M) compared to vehicle and HSA (*n* = 6). ***: significantly different with *P*_value_ < 0.01.

*In vitro* cytotoxicity study of SN38-HSA conjugates in comparison with those of free SN38 and irinotecan was assessed by MTT assay on HT-29 human colon cancer cells. As plotted in Figure 5, cellular viability was relatively decreased proportional to increasing SN38 (Figure 5(a)) and irinotecan concentrations (Figure 5(b)).

After 48 h incubation with samples the free SN38 IC_50_ was 0.12 ± 0.098 *μ*M, which is lower than IC_50_ of conjugates (1.12 ± 0.213 *μ*M for SN38-HSA-15 and 5.78 ± 0.195 *μ*M for SN38-HSA-60) (Table 2). Such obtained finding can be justified due to high stability of conjugates and SN38 slow release from conjugates. Moreover, conjugates demonstrated 3- to 14-fold superior cytotoxicity compared to that of irinotecan with IC_50_ value of 14.17 ± 1.273 *μ*M. Conjugate with less molar ratio (SN38-HSA-15) evoked 5.16-fold more cytotoxic effect than the SN38-HSA-60. Comparing cell viability results of SN38 conjugates having 1.6 *μ*M SN38, free SN38, irinotecan, vehicle, and HSA within 48 h incubation indicated that vehicle, HSA, and irinotecan with mentioned concentration did not cause toxicity (cell viability more than 90%). It was also revealed that the conjugate with higher molar ratio of SN38 conjugation had less toxicity compared to SN38-HSA-15 and free SN38 (Figure 5(c)).

### 3.6. Biodistribution Studies

**Figure 6:**
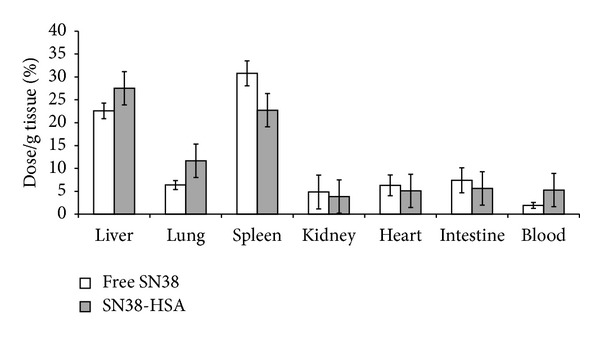
Biodistribution of free SN38 and SN38-HAS-15, 4 h after i.v. injection in balb/c mice.

As SN38-HSA-15 showed remarkable cytotoxicity and water solubility in comparison with SN38-HAS-60 so this conjugate has been chosen for* in vivo* studies. Evaluating free SN38 and SN38-HSA-15 distribution in different organs was investigated as though the resulting data was illustrated in Figure 6. According to these acquired results, it was evident that most of the free SN38 and SN38-HSA-15 were primarily collected by liver and spleen. These two organs are considered as the major organs possessing an active reticuloendothelial system (RES) [37]. The hepatic level of SN38 was 22.5% ± 1.70 and 27.5% ± 3.67 for free SN38 and SN38-HSA-15, respectively, while splenic level of free SN38 was 30.7% ± 2.73 and SN38-HSA-15 was 22.7% ± 2.84. Albumin conjugation of SN38 could not change the amount of distribution of SN38 in kidneys, heart, lung, and intestine (*P*_value_ > 0.05) compared to the free SN38. However, plasma level of SN38 was higher for HSA conjugate (5.2% ± 2.25) compared to free SN38 (1.9% ± 0.65). Together, biodistribution data clearly demonstrated that HSA conjugation as a drug delivery system was efficient by being able to improve the blood circulation time.

### 3.7. *In Vivo* Blood Cytotoxicity Tests

As shown in Table 3, treatment with SN38-HSA has no significant effect on blood cell counts (*P*_value_ > 0.05). Biodistribution study revealed that liver and kidneys are considered as the major organs which are vulnerable to drug accumulation. Thus, the biochemistry assays were employed to examine the effects of the conjugate on these organs. Findings demonstrated that the SN38 conjugate had no clinical signs of toxicity on liver and kidney (Table 3).

## 4. Discussion

The present study demonstrated the preparation process of HSA-SN38 conjugates and investigated their characteristics as well as their* in vitro* cytotoxicity,* in vivo* biodistribution, and blood toxicity. Such water-soluble conjugate was able to stabilize the SN38 structure in potent lactone ring form in physiologic condition [7]. Several different steps were required for both the SN38 activation towards HSA and the synthesis pathway. In order to achieve a selective conjugation of HSA to the aliphatic hydroxyl group of SN38, in the first step, the more active phenolic hydroxyl group was protected by a BOC group. In the second step, BOC-glycine was linked with a terminal SN38 aliphatic OH [7, 38]. Afterward, the BOC group was removed by addition of TFA and SN38-glycineate compound was linked with succinyl group. It is accurate to say that amines are more nucleophilic compared to that of hydroxyl groups. Therefore, the NH_2_ of glycine could react with succinic anhydride. Finally, conjugation between HSA and SN38 was achieved via the succinyl spacer group. In other words, carboxylic acid moiety is essential for reaction with amino groups of HSA and lack of this group has been fulfilled with succinyl spacer. As a result, succinyl spacer provided both a suitable site for amidation reaction and a favor distance between SN38 and HSA in aspect of the yield reaction. The average particle size of resultant conjugates was about 100 nm with appropriate polydispersity. The drug loading is proportional to the SN38: HSA ratio. Nevertheless, it has been shown that excessive attachment of hydrophobic molecules such as SN38 to a single albumin molecule can bring about significant decrease in the protein aqueous solubility [27].

Moreover, conjugates stability was examined in solutions with two different pH values (pH 5.2 and 7.4 to simulate acidic conditions of the cancer cells and physiologic conditions, resp.). Experimental data revealed that there was no difference in release profiles and pattern in different pH and various ratios of conjugates, although acidic condition led to increase the drug release from the conjugates due to the more susceptibility of the esteric bond to hydrolysis [27]. The delayed release of SN38 from the conjugates suggests their stability prior to administration and also their ability to release SN38 as an active compound after administration. The slow release was advantageous to maintain effective blood concentration of the drug which may necessitate less frequent drug administration [39]. The faster degradation in plasma as compared to pH 7.4 seems to indicate that in plasma the hydrolysis of the succinic ester bond takes place by both chemical and enzymatic mechanisms [40].

SDS-PAGE was performed to detect the molecular weight of SN38-HSA conjugates to reveal conjugates structures stability in comparison with the free HSA. Based upon obtained results it can be inferred that the procedures carried out to prepare the conjugates and the attachment of SN38 with the mentioned molar proportions did not alter the properties of HSA as the carrier protein.

*In vitro* cytotoxicity experiments proved that free SN38 and SN38-HSA conjugates had cytotoxic effect on HT-29 human colon cancer cell line. To release SN38 from conjugates, intracellular esteric bond hydrolysis was needed. This process is time consuming; thus cells were treated adequately with 48 h incubation; the free drug showed higher cytotoxicity than the conjugates. On the other hand, this effect may be related to the different uptake mechanisms of free drug with conjugates. The conjugate with higher drug loading (SN38-HSA-60) showed less cytotoxicity than the other conjugate. As it has been suggested before, the surface charge of the NPs can affect their cell permeation [41]. This conjugate exerted a higher negative amount of surface charge due to the involvement of more HSA amine groups in bonding with the linker. It can be concluded that high level of SN38 attachment on HSA can decrease conjugate cytotoxic potency as well as water solubility. In addition, the superior cytotoxic effect of conjugates compared with irinotecan was observed. The enhanced cytotoxicity effect obtained with SN38-HSA conjugates compared to irinotecan is evidently due to efficient cellular uptake of the conjugates into cells and protection of lactone structure inside conjugates whereas the properties of SN38 remain [7].

HSA is widely used as a carrier in the preparation of conjugates having antineoplastic properties. Interestingly, HSA is not only easily distributed in tumor tissues but also showed a long plasma half-life* in vivo *[26].* In vivo* biodistribution study is pivotal in monitoring the accumulation of a formulation in different organs [42]. Albumin conjugation of SN38 represented both higher and maintained level of blood concentration for up to 4 h in comparison to that of free SN38 that led to improvement of passive tumor targeting due to the EPR effect and consequently enhanced the antitumor efficacy [43, 44]. Biodistribution study indicated high accumulation of SN38 in liver and spleen for both free SN38 and SN38-HSA conjugation. This result is reasonable since these organs possess an active RES [45]. Additionally, blood cytotoxicity assays verified that SN38-HSA had no toxicity effect on liver, spleen, and blood cell counts as major organs which can be regarded as a substantial advantage over free SN38.

## 5. Conclusions

The aim of the present study was to prepare HSA conjugates of SN38. Our results introduced such conjugation as a valuable, stable, and soluble macromolecule successfully executing as a nanocarrier in overcoming potential attributable clinical limitations for SN38 application. The SEM images proved the average size of conjugates which was measured about 100 nm by laser light scattering. Furthermore, the drug content was investigated by means of drug loading. The conjugates were readily soluble in aqueous solutions and were stable in this medium before administration. Although, conjugates exhibited less cytotoxicity than free SN38 against HT-29 human colon cancer cells due to slow release rate, the superior cytotoxic effect of conjugates compared with irinotecan was observed. Moreover, biodistribution study indicated higher plasma level for conjugate compared to the free SN38. In spite of the high accumulation of conjugate in liver and spleen, no toxicity effect on these organs and also blood cell counts was found by blood cytotoxicity assays. In the current study conjugates presented both slow drug release with prolonged blood circulation time compared to free SN38. Taken together, it is assumed that the conjugation of SN38 with HSA improved the drug solubility so it can be potential candidate for clinical administration. Moreover, this conjugation improved the blood half-life of SN38 which might enhance the tumor targeting.

## Figures and Tables

**Table 1:** Size, PDI, zeta potential, and drug loading measurement of conjugates with two molar ratios of SN38/HSA.

Sample	Average size (nm)	PDI	Zeta potential (mV)	Drug loading (%w/w)
SN38-HSA-15	93.6 ± 7.34	0.27 ± 0.093	−14.3 ± 0.15	1.8 ± 0.09
SN38-HSA-60	109.1 ± 8.93	0.21 ± 0.084	−23.6 ± 0.37	4.4 ± 0.14

**Table 2:** IC_50_ values of free SN38 and SN38-HSA conjugates on HT-29 cell line within 48** **h of incubation.

Sample	IC_50_ (*μ*M)
Free SN38	0.12 ± 0.098
SN38-HSA-15	1.12 ± 0.213
SN38-HSA-60	5.78 ± 0.195
Irinotecan	14.17 ± 1.273

**Table 3:** Blood cell counts (RBC, WBC, and Hb) and biochemistry assays (AST, ALT enzymes, LDH, BUN, and Cr) in balb/c mice following 24 h treatment with SN38-HAS-15 versus control group. Values are presented as mean ± SD (*n* = 3).

Assays	Sample	Control	SN38-HSA-15
Blood cell counts	WBC (1000/*μ*L)	3.3 ± 0.611	3.1 ± 1.83
	RBC (mil/uL)	10.1 ± 1.40	9.8 ± 1.71
	Hb (g/dL)	14.5 ± 1.76	12.4 ± 2.18

Biochemistry	AST (IU/L)	291 ± 38.56	309.3 ± 29.31
	ALT (IU/L)	56.2 ± 6.08	53.6 ± 3.76
	LDH (IU/L)	1052 ± 97.52	1067 ± 122.2
	BUN (mg/dL)	53.5 ± 5.56	56.6 ± 7.88
	Cr (mg/dL)	0.5 ± 0.04	0.5 ± 0.02
